# Evaluating the effectiveness and feasibility of nurse-led distant and face-to-face interviews programs for promoting behavioral change and disease management in patients with diabetic nephropathy: a triangulation approach

**DOI:** 10.1186/s12912-020-0409-0

**Published:** 2020-03-12

**Authors:** Kana Kazawa, Kanae Osaki, Md Moshiur Rahman, Michiko Moriyama

**Affiliations:** Graduate School of Biomedical and Health Sciences, Hiroshima University, Kasumi 1-2-3, Minami-ku, Hiroshima, 734-8553 Japan

**Keywords:** Telenursing, Diabetic nephropathy, Face-to-face interview, Distance interview, Behavioral changes

## Abstract

**Background:**

We examined whether telecommunication-device-based distance interviews are inferior to face-to-face interviews in terms of facilitating behavioral changes and disease management in patients with diabetic nephropathy. We also examined the feasibility of a newly designed six-month telenursing program.

**Methods:**

This study represents a post-hoc analysis of data from a randomized controlled trial, in which we compared the efficacy of remote self-management education with that of direct education for patients with diabetic nephropathy. The participants were 40 company employees, who were randomly divided into two groups. Over 6 months, the intervention group (*n* = 21) received three distance interviews using a tablet computer. Meanwhile, the control group (*n* = 19) received three face-to-face interviews. In addition, both groups received biweekly nine telephone calls. A triangulation approach was used. We first compared the two groups in inferiority tests. Then, we analyzed data from semi-structured interviews with all participants and nurses, examining whether trusting relationships and motivation were developed, and the accuracy of the information exchanges. Further, for the intervention group, we also enquired about the overall operability of the telenursing device.

**Results:**

The completion rates for the program were 81.0 and 78.9% for the participants in the intervention and control groups, respectively. Both groups showed similar behavioral changes, and the participants verified the feasibility of the distance interviews. The participants in the intervention group felt that they understood the severity of their diseases and the necessity of self-management, and felt confidence in the nurses. On the other hand, their degree of behavioral change regarding self-monitoring was lower than that shown by the control group.

**Conclusion:**

Our findings show that both interview methods are effective for encouraging the adoption of self-management; further, in terms of taking medication and improving the main clinical indicators, we found that the distance method is not inferior to the direct face-to-face method. However, when considering long-term effects, based on the respective degrees of improvement in behavioral change, the direct method seems to be more effective.

**Trial registration:**

The trial was registered with the University Hospital Medical Information Network clinical trial registry (No. UMIN000026568) on March 15, 2017, retrospectively.

## Background

Diabetes mellitus is a significant health problem that affects approximately 10.1% of the population in Japan [1], and 422 million people worldwide [2]. It has the potential to cause chronic renal disease and is the most common indication for renal transplantation [3]. The prevalence of this condition is causing increasing concern for public-health and has motivated research into optimizing the management of diabetes care. Modern technologies such as telenursing (which concerns “the use of information and communication technology [ICT] to provide nursing care and conduct nursing practice at a distance” [4]) are considered essential tools for supporting patients with diabetes [2]. The inclusion of such technologies in health care has been receiving increased attention in recent decades, partly driven by the development of ICTs that may improve the management of patients with chronic diseases, including diabetes. In particular, it is widely recognized that telenursing can facilitate cost-effective delivery of patient education; this is because telenursing can reduce the number of hospital visits required [5], prevent complications [6], reduce mortality rates [7], and improve patients’ self-management behaviors [8]. However, there have been few attempts to compare telenursing’s effectiveness for supporting patients with diabetes with that for traditional face-to-face interview methods [9]. Notably, it has been suggested that patients show stronger motivation to receive self-management education and implement disease management when they receive face-to-face interviews with nurses than when they receive distance interviews [10]. In addition, only direct face-to-face contact enables actual tactile physical examination of patients; observing physical signs is particularly important for patients with severe diabetic nephropathy. Nevertheless, there are challenges associated with traditional face-to-face interviews, such as the cost or time required for nurses to travel to patients’ locations.

Distance interviews, conducted using newly developed ICT devices, are often emphasized as a means of avoiding the abovementioned obstacles associated with face-to-face interviews. This is because patients can undergo distance interviews in locations of their choosing (such as their homes or workplaces or any other convenient place) and can also feel greater convenient regarding the time of such interviews. However, distance interviews involve other challenges. For instance, patients can become nervous regarding operating tablets or computers, difficulties relating to technology and connection quality can arise, and the nurses who conduct the interviews require intensive training in related skills [11]. There have been many attempts to overcome these obstacles and improve telenursing for patients with chronic diseases, mainly through testing various telecommunication devices [12, 13]. However, although the popularity of telenursing is increasing, relatively few studies have compared direct face-to-face interviews with distance interviews in terms of the methods’ respective efficiency regarding engaging patients and facilitating behavioral change [14, 15].

The present authors previously evaluated the efficacy of a new distance interview method, involving tablet computer devices, in terms of its ability to facilitate behavioral change and disease management. Using a randomized controlled trial (RCT) approach, we compared a distance-interview group with a direct face-to-face interview group over 12 months (comprising a six-month intervention period, followed by 6 months of observation) [16]. The participants were patients with diabetes who had stage G3 nephropathy or worse; thus, these individuals had various complications and a high risk of acute aggravation, including cardiovascular complications. Consequently, the nurses who provided the education needed to accurately assess the participants’ physical conditions, and to teach the patients tailored self-monitoring techniques. Such nurse assessments and effective self-monitoring have critical implications for the early detection of clinical abnormalities, which is necessary for the timely implementation of remedial actions.

Although this previous RCT had a small sample size, we found that the two interview methods produced almost identical care effects, in terms of both behavioral changes and improvement in clinical indicators, at the 12-month follow-up (the two-sided 95% confidence interval for the difference in percent change was within approximately 10%). The main results of the entire 12-month study can be summarized as follows [16]: (1) In both groups, all self-management behaviors and clinical and psychological indicators improved; in addition, renal function was maintained. (2) The two groups showed comparable changes in self-management behaviors, with the exception of self-monitoring of blood pressure (BP) and body weight, for which the direct face-to-face method was more effective. (3) The groups showed comparable clinical and psychological indicators; however, patients in the direct face-to-face group showed significant improvement in serum creatinine, estimated glomerular filtration rate (eGFR), non-high-density lipoprotein cholesterol, BP, and quality of life (QOL) when compared with those in the distance method. (4) None of the patients initiated renal dialysis.

Some previous studies on telenursing for high-risk patients with diabetes have combined direct face-to-face interviews with distance interviews (via telephone or other telecommunication devices), but no studies have examined the establishment of trust relationships between nurses and patients, nurses’ motivation abilities, and the accuracy of health assessments in the context of distance interviews [17].

In the present study, we examined whether telecommunication-device-based distance interviews are inferior to face-to-face interviews in terms of facilitating behavioral changes and disease management in patients with diabetic nephropathy. We also examined the feasibility of a newly designed six-month telenursing program. This is performed by using a quantitative and qualitative triangulation approach.

## Methods

### Design

This research comprises a post-hoc analysis of the 12-month RCT described in the previous section [16]. We used a methodological triangulation design that involved both quantitative and qualitative methods; this allowed us to confirm our findings through considering the convergence of different perspectives [18, 19]. This strategy can compensate for difficulties interpreting results from small sample sizes, and can also afford the addition of participants’ opinions and impressions (which cannot be obtained through quantitative data); this means that more comprehensive results can be obtained. In addition, this strategy can increase the reliability of the findings, as it requires that information be confirmed using two or more measures [20].

First, we compared the distance-interview method’s effects on behavioral changes and clinical and psychological indicators with the direct face-to-face method’s effects on the corresponding variables. Second, feasibility was compared quantitatively, and then qualitatively.

### Participants and research procedure

We conducted this study in Japan, from October 2014 to February 2016. The intervention period was October 2014 to May 2015, and the follow-up period was April 2015 to December 2015. The study design and intervention protocols have been described in detail elsewhere [16]; the following is a brief description. Participants were recruited from approximately 200 branches of a private Japanese company. Among the company’s 350,000 employees, 180 met the following eligibility criteria: (i) having a proteinuria of ≥2+ or a proteinuria of 1+ and a hemoglobin A1c (HbA1c) of ≥7.0% (or a fasting blood sugar of ≥130 mg/dL) at a health check conducted in 2013 (an annual check for individuals aged 40 years or older), and (ii) diagnosed with type-2 diabetes. Of the total number of eligible employees (*n* = 180), only 40 consented to participate in the study because they were receiving health guidance by their family physicians, had no time to participate, and did not intend to change their lifestyle. The participants were randomly assigned to the intervention group (IG; *n* = 21) or the control group (CG; *n* = 19) (Supplementary File 1). Notably, this study excluded patients who had type-1 diabetes or gestational diabetes, had initiated dialysis, were scheduled for renal transplantation in the near future, were undergoing treatment for cancer, had a terminal illness, had cognitive impairment, and/or who had mental disorders. The required sample size was calculated based on the approach used in a previous study [21], with the standardized effect size (behavior change) = 0.50, two-sided α = 0.05, and β = 0.2. In this study, the estimated number of withdrawals was set at six people; the calculation consequently returned a value of 70 members in each group. Due to the limitation of budget and contractual agreements with the collaborative company, the extension was not allowed until the sample size was met. It was also difficult to ask other organizations for further collaboration to extend this study.

Before being randomized, the persons who consented to participate in the study were stratified by sex and age (≤ 59 years and ≥ 60 years), and according to the prevalence of tablet-device use in Japan [22].

### Program protocol

The participants underwent the abovementioned six-month program that we developed for examining self-management behavior [16]. The effectiveness of this program has already been documented [23]. The program was implemented by nurses who were trained in disease management. The study was implemented as follows:

1. The CG (received direct face-to-face interviews and intermittent telephone calls): From months one to three, individual direct face-to-face interviews of approximately 1 h in length were held once a month (three interviews in total) in a private room at the participants’ workplaces or in a public facility where the participants’ privacy could be protected. Biweekly follow-up calls (nine in total) were made via telephone; these calls involved providing education for patients, and were approximately 30 min in length, respectively. The education was provided based on evidence-based clinical guidelines for diabetes [24, 25] and for chronic kidney disease (CKD) [26]. CKD stage was assessed at the initial interview using laboratory data. Further, risk factors were identified using laboratory data, and by analyzing the participants’ existing treatments and information obtained from the physical examination and from evaluations of the participants’ lifestyles. The nurses explained the pathology and management of diabetic nephropathy using a guidebook developed by the researcher, and discussed goals for improving the participants’ lifestyles. Subsequently, the participants measured their BP, body weight, and blood glucose values regularly at home by themselves, recorded the results in a self-monitoring notebook and reported the values through video and phone calls. Then, they monitored changes in self-monitoring values, judged abnormalities and changed appropriate lifestyles accordingly. The nurses also assessed whether participants took medications, visited the clinic periodically, and followed the diet and exercise therapy they were recommended in order to achieve the goals they had defined with assistance from the nurses. The participants’ results for all variables were reported to the nurses every month. The guidebook, the self-monitoring notebook, and a monofilament (for foot care) were delivered to the participants by mail.
2. The IG (underwent distance interviews and only received intermittent telephone calls): This group underwent three distance interviews via a tablet computer in place of the three direct face-to-face interviews received by the CG. The tablet, featuring an application with instructions (explained below), was delivered to the participants by postal mail. The guidebook was included in the application, but a paper version was also delivered to the participants by mail, together with the self-monitoring notebook and foot care monofilament (similar to the CG).

### Telenursing system and nurses’ orientation

The IG participants used a tablet computer (iPad mini; iOS7.1.1), and the corresponding nurses used a desktop personal computer. A video providing education regarding how to self-monitor BP, weight, and edema on feet, and an education guidebook that described the pathology of diabetes and its recommended management in daily life (diet, exercise, medication, stress management, smoking secession, and alcohol-intake reduction) were installed on the tablet to allow the participants to perform self-learning. The device was equipped with a function that allowed nurses to mark the guidebook or enter figures on the screen in order to highlight explanations for the participants. A manual describing how to use the tablet and perform troubleshooting was prepared and delivered to the participants by postal mail.

Before commencing the interview, we asked each participant to check the telecommunication connection quality. If video or sound were interrupted during an interview, the nurse gave additional explanation via a mobile phone (cell phone). Prior to beginning the intervention, nurses received training in effective communication methods and means of performing physical evaluations using distance devices.

### Evaluation indicators and data-analysis procedure

#### Efficacy

(a) The efficacy of the participants’ self-management behaviors (diet, exercise, self-monitoring, medication) was analyzed using the model of behavioral change created by Prochaska and DiClemente (Table 1) [27]; responses were given using a five-point Likert scale. (b) Clinical indicators were eGFR, HbA1c, systolic and diastolic BP, and body mass index (BMI) values. (c) Psychological indicators were Self-Efficacy Scale for Health Behavior in Patients with Chronic Disease [28], and for two items concerning global QOL, which were sourced from the Japanese version of the World Health Organization Quality of Life scale [29].

**Table 1:** Self-management indicators

Stage	Diet	Exercise	Self-monitoring	Medication (frequency of behavior)
1 Precontemplation	● The participant does not intend to start dietary therapy or change current eating habits. ● The participant has no knowledge of dietary therapy.	● The participant does not intend to start physical exercise. ● The participant has no knowledge of exercise therapy (balance between exercise and rest).	The participant does not perform any self-monitoring.	The participant takes or injects medication when they remember it (approximately 1 to 2 days a month).
2 Contemplation	The participant is interested in dietary therapy and tries to understand, but has not started yet.	The participant is interested in exercise therapy (balance between exercise and rest) and tries to understand, but has not started yet.	The participant is interested in monitoring and tries to understand, but has not started it.	The participant takes or injects medication 1 to 2 days a week.
3 Preparation	● The participant understands the problems with current eating habits. ● The participant has started to take action.	The participant has started to perform some exercise (balance between exercise and rest).	The participant can measure and record the parameters properly.	The participant takes or injects medication 3 to 4 days a week.
4 Action	● The participant understands the instructed food intake. ● The participant can plan improvements and act with advice from others.	● The participant understands the proper amount and timing of physical exercise. ● The participant can plan improvements and act with advice from others.	● The participant understands the monitoring instructions of the physician. ● The participant can analyze the measurement and modify treatment based on advice from others.	The participant takes or injects medication 5 to 6 days a week.
5 Maintenance	● Continuing dietary therapy ● The participant can plan to improve the diet and act autonomously.	● Continuing exercise therapy ● The participant can plan to improve exercise and act autonomously	● Continuing monitoring ● The participant can analyze the measurement and modify treatment autonomously.	● The participant takes or injects medication almost every day. ● The participant can take the necessary action when a dose is missed.
6	Participant not in the program for medical reasons (fasting, parenteral nutrition)	Physical exercise is prohibited	The participant cannot perform self-monitoring (e.g., cervical vertebral injury)	Not prescribed

For indicators (a) to (c), the percent change over 6 months [(value at 6 months - value at enrollment)/value at enrollment × 100] was calculated. We then compared the two groups to evaluate whether the results for the IG (distance method) were inferior to those of the CG (direct face-to-face method). The following criterion was used to compare the characteristics of these two methods and assess non-inferiority: when the two-sided 95% confidence interval for the between-group difference in percent change was within approximately ±10%, the effect of the two methods was considered to be comparable. Through statistical analysis, the percent change over 6 months was tested for normality. In addition, analysis of covariance was performed for each indicator, using data at enrollment as the covariates and each group as the independent variable.

#### Feasibility

A Likert-scale questionnaire, for which the responses ranged from “strongly agree” to “strongly disagree,” was administered to the participants at the end of the intervention. Semi-structured telephone interviews were also conducted with all nurses and participants. The interview guide comprised the following questions (listed in the order of question for the participant/question for the nurse): “Did the interview motivate you to commence/commit to self-management?”/“Do you think you successfully motivated the participant?”; “Did you trust the nurse?”/“Do you think you successfully developed a trusting relationship with the participant?”; “Did you willingly implement action plans (change your lifestyle)?”/“Do you think you successfully motivated the participant to change his/her behavior?”; “Do you think both you and the nurse assessed your body condition properly?”/“Did you have difficulty assessing the participant?”; “What was your experience operating the iPad? Did you have any difficulties?” After obtaining the answers to these questions, the researcher asked further in-depth questions if clarifications were considered necessary.

All participants’ interviews were audio-recorded, and transcripts were created of each. The relevant contents were extracted according to the evaluation categories in Table 4.

#### Integration

Finally, we interpreted and compared the quantitative and qualitative results.

This paper adhered to the SRQR recommendations.

## Results

### Completion rate and participants’ characteristics

As shown in Supplementary File 1, during the period between the signing of the informed consent form and measurement of baseline data, three participants from the IG and two from the CG decided to withdraw from the study. Further, during the intervention, and additional participant from the IG and two from the CG withdrew because of lost to follow-up and being discontinued intervention. Thus, the end of program analysis was conducted on 32 participants (IG: *n* = 17, completion rate = 81.0%; CG: *n* = 15, completion rate = 78.9%). Table 2 shows the results of a comparison of the two groups’ baseline characteristics. This shows that participants’ characteristics did not differ between the two groups, but that BMI was significantly lower in the CG (Student’s t-test, *p* = .013).

**Table 2:** Baseline characteristics of the participants

Variable	Intervention group (*n* = 18)		Control group (*n* = 17)		*P*-value^†^
	n	Mean ± SD	n	Mean ± SD	
Age (year)	18	59.4 ± 9.1	17	57.6 ± 6.9	0.502
Sex, n (%) Men	18	16 (88.9)	17	17 (100)	0.157
Duration of diabetes (years)	17	11.8 ± 10.1	17	9.2 ± 9.2	0.451
eGFR (ml/min/1.73 m^2^)	17	54.7 ± 14.7	16	55.2 ± 17.8	0.927
HbA1c (%)	17	7.7 ± 1.6	17	7.6 ± 1.3	0.860
Systolic BP (mmHg)	18	138.3 ± 13.6	17	146.8 ± 16.6	0.104
Diastolic BP (mmHg)	18	84.2 ± 9.8	17	87.9 ± 9.0	0.258
BMI (kg/m2)	18	30.1 ± 4.5	17	26.2 ± 4.2	0.013

*Definition of abbreviations*: *eGFR* estimated glomerular filtration rate, *HbA1c* hemoglobin A1c, *BP* blood pressure, *BMI* body mass index

^†^Differences were evaluated using Student’s t-test. However, Chi-square test was used to compare differences in gender between the two groups

### Quantitative comparison between the results for the distance method and the direct face-to-face method

Table 3 presents the results of the inferiority test. Here, indicators that showed comparable efficacy between the two groups were medication, HbA1c, diastolic BP, BMI, and self-efficacy-scale score. We noted that the CG showed a larger improvement in implementation of self-monitoring when compared to the IG. When inter-group comparison of the percent changes was performed, an interaction was noted with regard to implementation of exercise; therefore, the exercise data were not assessed in analyses of covariance.

**Table 3:** Comparison of efficacy (difference in percent change) in indicators between the groups

Variable	Intervention group (*n* = 17)										Control group (*n* = 15)										Adjusted mean difference between the groups (95% CI)^b^	
	n	Baseline			6 months			Mean percent change (%)^a^			n	Baseline			6 months			Mean percent change (%)^a^				
Self-management behaviors																						
Dietary stage	17	2.7	±	0.6	3.9	±	1.0	54.9	±	52.6	15	2.4	±	0.8	3.9	±	0.5	90.0	±	99.2	−15.78	(−56.16 to 24.60)
Exercise stage	17	2.9	±	0.7	3.8	±	1.0	29.9	±	31.3	14	2.7	±	1.1	3.6	±	0.9	48.9	±	54.4	With interaction	
Self-monitoring stage	17	2.4	±	0.6	4.1	±	0.7	81.4	±	51.7	15	2.0	±	1.3	3.9	±	0.8	166.1	±	138.6	−50.29	(−93.97 to − 6.61)
medication/injection stage	17	4.4	±	0.6	4.9	±	0.3	13.0	±	19.9	14	4.9	±	0.3	5.0	±	0.0	1.8	±	6.7	−3.50	(−8.92 to 1.92)
Clinical indicators																						
eGFR (ml/min/1.73m^2^)	12	50.3	±	12.1	53.2	±	13.4	5.9	±	8.7	8	61.2	±	20.2	62.4	±	23.3	1.1	±	14.5	5.51	(−6.05 to 17.06)
HbA1c (%)	16	7.7	±	1.6	7.3	±	1.4	−4.2	±	6.5	12	7.8	±	1.1	7.2	±	1.0	−7.2	±	8.9	2.75	(−2.95 to 8.45)
Systolic BP (mmHg)	17	138.2	±	14.0	133.8	±	10.9	−2.7	±	7.6	15	147.7	±	17.2	127.3	±	11.9	−13.0	±	10.7	6.44	(1.30 to 11.58)
Diastolic BP (mmHg)	17	84.5	±	10.0	80.1	±	7.6	−4.5	±	10.8	15	87.8	±	9.5	79.3	±	6.3	−8.6	±	13.3	1.04	(−4.88 to 6.96)
BMI (kg/m^2^)	17	29.7	±	4.4	29.1	±	4.5	−2.2	±	2.3	15	26.0	±	4.3	25.6	±	4.0	−1.5	±	3.2	−0.21	(−2.40 to 1.98)
Psychological indicators																						
Self-efficacy score^c^	11	70.4	±	11.0	74.4	±	9.8	6.5	±	9.1	13	75.0	±	9.9	78.5	±	9.1	5.3	±	10.3	−1.38	(−8.52 to 5.76)
QOL score^d^	11	2.78	±	0.56	4.27	±	0.56	58.5	±	32.7	13	2.35	±	0.90	3.81	±	0.72	82.4	±	63.8	0.84	(−23.86 to 25.5)

There were no significant differences in the baseline of self-management indicators between the two groups

*Definition of abbreviations*: *eGFR* estimated glomerular filtration rate, *HbA1c* hemoglobin A1c, *BP* blood pressure, *BMI* body mass index, *QOL* quality of life

^a^Mean percent change (%) = (6 months - Baseline) /(Baseline * 100)

^b^Analysis of covariance adjusted for baseline data

^c^Self-efficacy score: 24–96 points, with a higher score indicating greater self-efficacy

^d^QOL score: 1–5 points, with a higher score indicating better QOL

### Comparison of changes in self-management behaviors

Both groups showed similar improvements in all self-management behaviors, from the contemplation stage (see Table 1) at baseline, to preparation, to the action stages at the end of the program. The mean percentage change was larger in the CG than in the IG.

### Comparison of changes in clinical indicators

Renal function (eGFR) did not change in either group; meanwhile, HbA1c improved in both groups, with the CG showing larger improvement. Both groups showed changes in systolic and diastolic BP, with the CG showing larger changes. BMI showed a very small decrease in both groups.

### Comparison of changes in psychological indicators

Self-efficacy and QOL scores improved in both groups. The CG showed a larger increase in QOL.

### Qualitative analysis of participants who did not show behavioral changes

We analyzed the characteristics of four participants in the IG and two in the CG who failed to reach stage 4 for dietary-therapy-related self-management behaviors (action stage; see Table 1), which indicated that the nurses failed to motivate them in this regard. Their common characteristics were: 1) they were at stage 3 (preparation) or lower for exercise and self-monitoring at the end of the program period; and 2) they were obese (BMI range: 26–40). By the end of the program, five of these six participants had not developed self-monitoring habits, and only two of the participants from the IG, and none from the CG, achieved the weight-loss targets.

The nurses who interviewed these participants reported that the participants did not adopt appropriate lifestyles or monitor data changes in their health parameters; this suggests that these participants were not interested in their physical signs or in controlling their conditions.

### Comparison between the distance method and the direct face-to-face method from a feasibility perspective

**Fig. 1:**
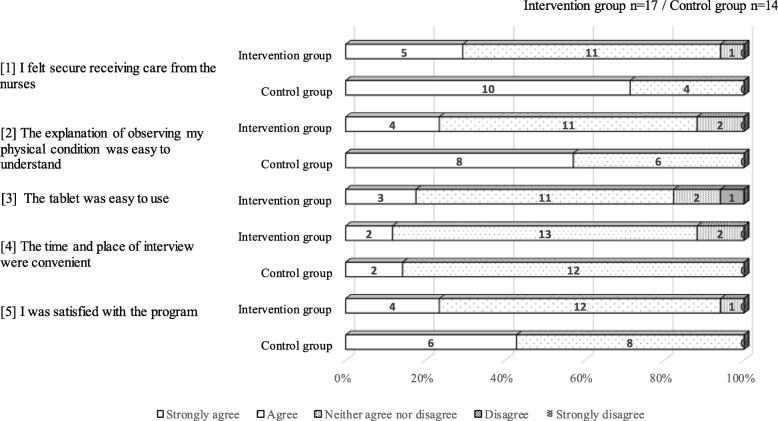
Feasibility evaluation by the participants from both groups. One member of the control group did not return the questionnaire. There were no differences between the two groups regarding the participants’ evaluations

For the two groups, participants’ opinions regarding the nurses’ motivating ability and the development of trust between participants and nurses, their satisfaction with the assessments and monitoring they received, their perceptions regarding the convenience of the interview method, and their overall satisfaction with the interview method were compared. Further, the IG’s opinions concerning the operability of the devices were also evaluated (Fig. 1 and Table 4).

**Table 4:** Feasibility evaluation of distance interview method by participants and nurses

Categories	Distance interview method
Development of trust relationship, engagement, and motivation for change	***Participants*** - They understood the severity of their diseases and necessity of self-management, but they felt difficulties to see the methods of self-monitoring of blood pressure measurement and palpating edema by screen (without hand-on demonstration). - They felt secure and firmly attached to the nurses and adequately guided. - Delayed voice transmission and small screen hindered communication. - Because of the small screen, one was not sure if the nurse understood him. ***Nurses*** - Physical and facial expression technique (such as widely nodding the head, lowering voice tone, and consciously making interpose) were needed to clearly communicate. - It was difficult to build a trust relationship without having direct eye contact and touching body parts such as foot care. - One felt insecure in constraining the patient to behavior modification because she was not sure of the participant’s facial expression.
	***(Reference)*** ***Participants in direct face-to-face interview method*** - It was emotionally easy to communicate, and they felt very close to the nurses. - They felt easy to understand the methods of self-monitoring because the nurses demonstrate directly to them. ***Nurses*** - All nurses, except one, felt no differences in both groups in behavior modification as long as nurses followed motivation interview techniques. - Physical contact in a direct face-to-face interview made better engagement and trust relationships.
Getting accurate information needed for health assessment	***Participants*** *-* One felt unsure that the nurse understood his physical condition and facial expression. ***Nurses*** - It was difficult to see the participants’ lab data sheets. - It was difficult to grasp the whole body (overall impression) at a glance by a small screen. - Visual inspection of the images without body palpation could not allow the nurses to assess the participants’ conditions.
	***(Reference)*** ***Participants in direct face-to-face interview method*** - It was easy to share detailed information.
Operability of the device for the distance interview	***Participants*** - For tablet computer users, operating the tablet was not difficult, but for the first user or older user, it took time to get used to it. (An operation manual was useful, and the nurse explained before starting was helpful.) - (Some had reduced visual acuity associated with aging and/or diabetic retinopathy, but no one had difficulty of watching the screen.) - Clarity of images or sound depended on transmission condition of participants’ homes. Some participants had difficulties in using the device and needed to use additional devices such as earphones and bright lightning. ***Nurses*** - It was difficult to teach the participants how to use it. However, nurses prepared an easy instruction and troubleshooting manual.
Privacy protection	***Participants in both groups*** - Both felt protected (because the nurses explained to have an interview in a private room.)
Additional benefits	***Nurses*** - Nurses were able to observe inside of participants’ houses. Participants were open to show their homes. Family members, such as a spouse and children, could easily join the meeting. Therefore, family members learned and shared the education.

The participants from both groups generally gave positive responses to all questions. However, more participants from the CG reported feeling confidence in their nurses, well-assessed, and satisfied (Fig. 1). Supporting this result, as shown in Table 4, most IG participants and all nurses reported confidence in the method and a trusting relationship with their patient/nurse, but both sides mentioned needing to take additional actions to address issues with the device regarding communication and images. To facilitate communication, nurses needed to make expressive gestures (such as exaggeratedly nodding their heads) and change their tone of voice. One nurse reported that she did not have confidence in her ability to determine changes in participants’ motivation through distance interviews. Further, some patients in the IG reported experiencing difficulties understanding the self-monitoring methods. Regarding the accuracy of the clinical observations (physical signs and symptoms), the nurses for the IG participants experienced difficulty performing detailed assessments of the participants’ physical conditions. Unexpectedly, virtually all of the participants (with the exception of two from the IG) expressed affirmative opinions regarding the convenience and location of the interviews. Nurses reported that the distance method (smart tablet use) allowed them to observe a great deal of the participants’ living environments and to invite significant others such as family members to assist, which they felt was effective (for the direct face-to-face interviews, for security reasons, the nurses met participants at public spaces, such as a community centers and company meeting rooms).

Finally, the IG reported that tablet operability depended on transmission quality, which was sometimes unstable; however, 14 members (82.4%) of the IG felt that it was easy to operate.

## Discussion

### Integration of quantitative and qualitative results

#### Efficacy

In this study, we examined, by integrating quantitative and qualitative data, whether a distance interview method is inferior to a direct face-to-face method in terms of facilitating behavioral changes and disease management in patients with diabetic nephropathy. Quantitative analysis showed that all indicators improved in both groups, with dietary stage, medication, HbA1c, diastolic BP, BMI, self-efficacy score, and QOL score showing comparable changes, respectively, in the two groups. However, although both groups showed improved behavior changes regarding self-monitoring, a greater improvement was found for the CG. Self-monitoring, which involves setting goals for oneself and performing daily evaluations, is a primary method of implementing behavior change and achieving continuity. Our findings show that both interview methods are effective for encouraging the adoption of self-management; further, in terms of taking medication and improving the main clinical indicators, we found that the distance method is not inferior to the direct face-to-face method. However, when considering long-term effects, based on the respective degrees of improvement in behavioral change, the direct method seems to be more effective.

These findings were supported by qualitative data. Many participants in the distance method group reported that they found the self-monitoring technique difficult to understand without hands-on demonstration. Additionally, we analyzed the characteristics of the participants who did not show any behavioral change; all had low adherence to self-management, except for drug therapy, and were obese. Obesity causes abnormal glucose tolerance, abnormal lipid levels, and hypertension, and also increases the risk of cardiovascular disease and death [30]. The nurses felt that high-risk patients with diabetes and obesity had trouble understanding the associated risk factors (such as edema and high blood pressure), and the nurses consequently had difficulty motivating the participants to perform self-monitoring. This was because the nurses could only perform physical assessment through observations and interviews via the tablet. Encouraging patients to learn proper self-monitoring methods early in the intervention leads to an understanding of physical risks, a sense of crisis, and increased motivation. Therefore, we believe that at least one face-to-face interview, in which detailed health status is assessed and motivation is provided, may be necessary for such patients.

#### Feasibility

The completion rate for both groups was approximately 80% and, for the remaining 20%, the reasons for withdrawal were not related to convenience. This indicates that once participants are successfully engaged, they are likely to complete the program. This indicates that the feasibility of the approach was of an acceptable level.

Although the IG provided a high overall evaluation, more participants and nurses from the CG reported feeling confidence in the method, satisfaction with the physical condition assessment, and overall satisfaction, all of which were consistent with the interview data. In the IG, the nurses needed to learn additional skills to compensate for the lack of direct observation and ability to perform physical examinations. A means of helping nurses obtain such skills would be performing step-by-step demonstrations using tablets.

Development of trust relationships, engagement, and motivation to effect behavioral changes: Participants in the IG felt confidence in their nurses. However, some participants in this group reported that they were unable to discern the nurses’ facial expressions on the tablet’s small screen, and that voice transmission was occasionally interrupted. With respect to the motivation for behavioral change induced by the nurses, both groups showed behavioral changes and improved self-management indicators. Self-monitoring, however, improved to a greater degree in the CG than in the IG. In addition, the participants in the CG reported being better able to understand the self-monitoring methods when compared to those in the IG. We installed on the telenursing tablet a video that explained self-monitoring, but the participants seldom watched it, and the nurses were unable to fully confirm the participants’ understanding or implementation of self-monitoring.

Obtaining accurate information needed for health assessments: As mentioned above, the nurses felt that it was difficult to motivate patients based on detailed physical risk assessment. For patients with diabetes, improving self-monitoring ability and the motivation to perform self-monitoring and to adhere to treatment are essential components for the adoption of self-management behaviors that improve the health condition and QOL [31–33]. In order to achieve this goal through the medium of telenursing, improvement in telecommunication is necessary, as this would allow nurses to assess patients’ physical conditions, and would also allow the patients to gain an understanding of their physical status and the effects of self-management [34].

Operability of the device for distance interviews: A small number of participants reported that they could not satisfactorily send/receive sound and/or images, even when they tested the telecommunication connection quality in advance. Some participants had reduced visual acuity associated with aging or diabetic retinopathy, but none had visual-field loss or severe visual disturbance. In an effort to address difficulties regarding using the device, we post-mailed to the participants, before the interviews had commenced, a manual that provided instructions regarding how the tablet should be operated. Most participants followed the instructions in the manual and did not experience any related issues when participating in the interviews. However, one participant had difficulty operating the tablet, and the nurse provided instructions regarding its operation via telephone. Based on these findings, we believe that patients with visual impairment should have face-to-face interviews instead of distance interviews, because such patients cannot see clearly images or the nurse when using the tablet device. Moreover, patients who are not accustomed to telecommunications devices, such as older patients, may also need special consideration [35, 36]; such patients should receive face-to-face interviews if they have difficulty operating the tablet, or family members could be trained in its operation so that they can provide assistance (i.e., the family may participate in the interview or operate the tablet on behalf of the patient) [37]. In the present study, the tablets were only used for the transmission of images and voice. The quality of the telecommunication devices used in telenursing has an impact on the safety and effectiveness of the health care provided [38]. The development of new devices that facilitate communication with and the education and empowerment of patients would be desirable; in particular, functions for visualizing data for self-management or monitoring would be a valuable addition [39–41].

The above findings suggest that, for patients who do not have specific characteristics that make remote interventions difficult, distance interviews may be a feasible substitute for face-to-face interviews.

### Limitations

This study featured a small sample size and, to address this, we applied a triangulation approach; however, interpretation of the results remained limited. Therefore, along with strengthening the motivation of target populations to participate in such research, future large-scale trials in this area should seek to obtain further evidence of the effects of the distance interview method. Additionally, in this study almost 80% of the employees who met the inclusion criteria refused to participate. While this indicates a need to develop a distance-education tool that is convenient for such individuals, it also implies that, in order to achieve effective recruitment. For example, it seems necessary that the nurses explain to the participants their risk of aggravating the disease and conducts motivation interviews.

### Implications for practice

In developed countries such as Japan, health systems are now focusing on chronic illnesses such as lifestyle-related diseases and cancer. Disease-management programs, especially those for preventing people with diabetic nephropathy requiring dialysis, are a high priority. This distance-education method, which involves the use of ICT devices, facilitates the provision of health care to people all over the world, regardless of location and time constraints.

## Conclusion

Results suggest that our distance interview program can improve the health status of patients with diabetic nephropathy, provided they do not have specific characteristics that hinder remote intervention. However, distance interview did not show the same levels of changes as the direct interview showed. The present study also revealed the feasibility of distance interviews. In the future, it will be necessary to improve this program and tool with the aim of educating patients on self-management techniques and of facilitating the exchange of accurate information for health assessment between nurses and patients.

## Supplementary information

**Additional file 1.** Participants flow chart.

## Data Availability

The datasets generated and analyzed during the current study are not publicly available because of research participants’ privacy.

## Abbreviations

BMI: Body mass index
BP: Blood pressure
CG: Control group
CKD: Chronic kidney disease
eGFR: Estimated glomerular filtration rate
ICT: Information and communication technology
IG: Intervention group
QOL: Quality of life
