# Supplementary material for: Evaluating the effectiveness and feasibility of nurse-led distant and face-to-face interviews programs for promoting behavioral change and disease management in patients with diabetic nephropathy: a triangulation approach
Source: BMC Nurs. 2020 Mar 12;19:16. doi: 10.1186/s12912-020-0409-0 (PMC7068973; doi:10.1186/s12912-020-0409-0)
Supplement: Supplementary file 1 — Additional file 1. Participants flow chart. [file 12912_2020_409_MOESM1_ESM.docx]

Analysed (n = 15)

Analysed (n = 17)

Assessed for eligibility (n = 180)

Not consented (n = 140)

Declined to participate (n = 140)

Include who was not able to contact

Consented to participate & Randomized (n = 40)

**Control Group (Direct face-to-face Interview)**

**Intervention Group (Distance Interview)**

Allocated to intervention (n = 19)

**Received allocated intervention (n = 17)**

Did not receive allocated intervention

(reason; lack of time) (n = 2)

Allocated to intervention (n = 21)

**Received allocated intervention (n = 18)**

Did not receive allocated intervention (reasons; lack of time and hospitalization) (n = 3)

6 months

## Program started

Discontinued intervention (reason; onset of cancer) (n = 1)

Lost to follow-up (reason; lost contact) (n = 1)

Discontinued intervention (reason; refusal to change lifestyle) (n = 1)

## Program ended

Supplementary File 1 　Participants flow chart
